# Differential Sympathetic Activation of Adipose Tissues by Brain-Derived Neurotrophic Factor

**DOI:** 10.3390/biom9090452

**Published:** 2019-09-05

**Authors:** Qi Zhu, Xian Liu, Bradley J. Glazier, Kristen N. Krolick, Shangyuwen Yang, Jingyan He, Chunmin C. Lo, Haifei Shi

**Affiliations:** 1 Department of Biology, Miami University, Oxford, OH 45056, USA; 2 Department of Biomedical Sciences, Heritage College of Osteopathic Medicine, Diabetes Institute, Ohio University, Athens, OH 45701, USA

**Keywords:** white adipose tissue, brown adipose tissue, energy homeostasis, lipolysis, adrenal hormones, sympathetic activity, denervation

## Abstract

Centrally administered brain-derived neurotrophic factor (BDNF) decreases body adiposity beyond what can be accounted for by decreased food intake, implying enhanced lipid metabolism by BDNF. Consistent with this notion, intracerebroventricular (icv) injection of BDNF in rats increased the expression of lipolytic enzymes in white adipose tissues (WAT) and increased circulating concentrations of lipolytic products without changing the levels of adrenal gland hormones. This suggests that central BDNF-induced lipid mobilization is likely due to sympathetic neural activation, rather than activation of the adrenocortical or adrenomedullary system. We hypothesized that BDNF activated sympathetic innervation of adipose tissues to regulate lipolysis. Rats with unilateral denervation of interscapular brown adipose tissue (BAT) and different WAT depots received icv injections of saline or BDNF. Both intact and denervated adipose tissues were exposed to the same circulating factors, but denervated adipose tissues did not receive neural signals. Norepinephrine (NE) turnover (NETO) of BAT and WAT was assessed as a measure of sympathetic activity. Findings revealed that central BDNF treatment induced a change in NETO in some but not all the adipose tissues tested. Specifically, greater NETO rates were found in BAT and gonadal epididymal WAT (EWAT), but not in inguinal WAT (IWAT) or retroperitoneal WAT (RWAT), of BDNF-treated rats compared to saline-treated rats. Furthermore, intact innervation was necessary for BDNF-induced NETO in BAT and EWAT. In addition, BDNF increased the expression of lipolytic enzymes in both intact and denervated EWAT and IWAT, suggesting that BDNF-induced WAT lipolysis was independent of intact innervation. To summarize, centrally administered BDNF selectively provoked sympathetic drives to BAT and EWAT that was dependent on intact innervation, while BDNF also increased lipolysis in a manner independent of intact innervation.

## 1. Introduction

White adipose tissues (WAT) store surplus energy primarily as triglycerides in adipocytes, and stored triglycerides are hydrolyzed through lipolysis to release free fatty acids and glycerol when energy needs cannot be met by circulating fuels or other stored fuels [1]. Brown adipose tissue (BAT) contributes to cold-induced thermogenesis in mammals, including adult humans, via activating uncoupling protein 1 (UCP1), which disassociates the respiratory chain from ATP production and ultimately dissipates energy as heat, a process that can be activated physiologically or pharmacologically [2]. The sympathetic nervous system (SNS) regulates lipid metabolism, which is involved in both lipolysis and thermogenesis, beginning with the principal postganglionic sympathetic neurotransmitter norepinephrine (NE) binding to β3-adrenergic receptor (β3-AR) located on adipocyte membrane [3].

Central nervous system (CNS) neurochemicals are involved in the control of sympathetic drive to WAT and BAT [1]. Brain-derived neurotrophic factor (BDNF), acting through its receptor tropomyosin-related kinase B (TrkB), is a neurochemical that regulates energy balance via suppressing energy intake and increasing energy expenditure [4]. Administration of BDNF into the CNS increases multiple aspects of whole-animal energy expenditure, including metabolic rate, oxygen consumption, heat production, and body temperature [5,6,7,8], likely due to the stimulatory effects of BDNF on sympathetic pathways regulating WAT lipolysis and BAT thermogenesis. Genetic mouse models with low expression of BDNF [9,10] or its receptor TrkB [11] exhibit reduced energy expenditure, which leads to obesity. Although it has been suggested that BDNF regulates energy metabolism via a close interaction with the SNS to increase fuel mobilization and energy expenditure, only a few in vivo studies have examined the effect of BDNF treatment on sympathetic activity in BAT. Specifically, in BAT, BDNF increases *Ucp1* mRNA level and UCP1 protein expression [6], heightens NE turnover (NETO) [8,12], and enhances thermogenesis [13]. It is currently unclear whether BDNF affects sympathetic activity in WAT, and if BDNF influences lipid metabolism in a similar manner among different types of adipose tissues. To our knowledge, there has been no comprehensive investigation into whether BDNF regulates lipid metabolism of various adipose tissues via sympathetic drive. We hypothesized that centrally administrated BDNF differentially induced sympathetic activity of WAT and BAT to regulate energy metabolism.

Two different approaches were used to assess sympathetic activity of adipose tissues in this study. The first approach was to measure NETO as an indicator for sympathetic drive using a tyrosine hydroxylase inhibitor, α-methyl-p-tyrosine (αMPT), which inhibits NE biosynthesis. The measurement of NETO determines sympathetic drive based on the depletion rate of NE release [14], which is independent of tissue response to NE or NE metabolism/clearance, and thus is used to assess sympathetic drive to target tissues [15]. The second approach was to quantify mRNA and protein levels of molecular markers related to sympathetic activity and sympathetic functions of adipose tissues using quantitative PCR and western blot analysis. Additionally, to exclude potential effects of circulating factors and prove effects of local innervation, unilateral denervation of WAT and BAT was performed while the innervation of contralateral matched adipose depots were kept intact to serve as within-subject controls. Both intact and denervated adipose tissues within each animal were exposed to the same circulating factors, but the denervated adipose tissues did not receive the neural signals that their contralateral intact adipose tissues did. We tested whether centrally administered BDNF induced elevated NETO and increased expression of SNS-related genes and proteins in individual WAT and BAT, compared to the control saline treatment; and if such regulation was reliant on intact innervation of WAT and BAT. Findings from this study provide biochemical and molecular insights into central BDNF regulation of lipid metabolism in BAT and WAT via the SNS, and determine whether such regulation is dependent on intact innervation of WAT and BAT.

## 2. Materials and Methods

### 2.1. Animals and Experimental Design

Two cohorts of adult (14–18 weeks-old) male Long-Evans rats (Envigo, Indianapolis, IN, USA) were used. All rats were single housed in a temperature- (22 ± 2 °C) and light- (12:12 light:dark; lights on at 0200 h and lights off at 1400) controlled vivarium. Standard rodent chow (Purina Rodent Chow No. 5001, St. Louis, MO, USA) and water were provided ad libitum unless specified on the testing days. All surgeries were performed when rats were under isoflurane anesthesia. All procedures were approved by the Institutional Animals Care and Use Committee of Miami University (protocol #947 approved on 5 January, 2016) and were conducted in compliance with the Guide for the Care and Use of Laboratory Animals as adopted by the National Institutes of Health.

The first cohort of twenty rats was used to test the effects of BDNF on whole-animal lipid mobilization. These rats received third ventricle cannula implantation surgeries. On testing days, body weights and body compositions were measured, and food was removed 4 h prior to lights-off [16]. Right before the onset of the dark, each rat received via intracerebroventricular (icv) injection either 1 µL 0.9% sterile saline or 0.3 µg recombinant BDNF (Millipore, Temecula, CA, USA) in 1 µL saline, the minimal dose of BDNF that is effective at suppressing feeding of adult male rats [16]. Food intake, body weights, and body composition were monitored 24 h after injections. After rats had fully recovered from icv saline or BDNF injections when their food intake and body weights were similar to their pre-experiment levels, they received another icv injection of either saline or BDNF, and were euthanized 6 h later. Trunk blood was collected, and plasma samples were stored at −80 °C for measurement of circulating lipolytic products and adrenal gland hormones. Adipose tissues were collected, rapidly frozen and stored at −80 °C until protein extraction for assessing expression of lipolytic enzymes using western blot analysis.

The second cohort of forty rats was used to test if BDNF affects adipose lipolysis via innervation. These rats received cannula implantation and unilateral adipose tissue denervation surgeries. On testing days, rats received icv injections of saline or BDNF, then were injected intraperitoneally (ip) with either vehicle or αMPT. Six hours after icv injections, the rats were euthanized, and intact and denervated BAT and WAT were collected, frozen, and stored at −80 °C until catecholamine extraction for measuring NE content to determine NETO, and extraction for total RNA and protein for analyzing gene and protein expression using quantitative PCR and western blot analysis respectively.

### 2.2. Cannula Implantation

Guide cannulae (22-gauge, Plastics One, Roanoke, VA, USA) aimed at the third ventricle were stereotaxically implanted into the rats as described previously [16]. Briefly, the rats were placed in a stereotaxic apparatus (David Kopf Instruments, Tujunga, CA, USA). The skull was exposed with lambda and bregma at the same vertical coordinate. A cannula was positioned on the midline from the dural meninge, and 2.2 mm anteroposterior and 7.5 mm dorsoventral with respect to bregma, which targeted at the third ventricle [17]. The guide cannulae were secured to the skull with mounting anchor screws (Plastics One) and dental acrylic resin BCA powder (Ortho-Jet^TM^). A removable obturator (Plastics One) was inserted into the guide cannula to cover its opening throughout the experiment, except when it was removed for injections.

### 2.3. Unilateral Denervation

Some adipose depots present bilaterally in the body and can be unilaterally denervated. To exclude effects of circulating factors, the left or right side of interscapular BAT, gonadal epididymal WAT (EWAT), subcutaneous inguinal WAT (IWAT), and visceral retroperitoneal WAT (RWAT) were unilaterally denervated with contralateral same type adipose tissues having intact innervation and serving as within subject controls, according to the established protocol [15,18,19]. For BAT denervation surgery, intercostal nerves existing in two major bundles that unilaterally innervate each side of BAT lobes were severed at two locations, with small nerve pieces removed between the cuts to prevent reconnection. Care was taken not to damage the Sulzer’s vein. For EWAT denervation surgery, EWAT and the testis were gently pulled from the peritoneal cavity. A drop of 1% toluidine blue dye was applied onto EWAT to facilitate visualization of the nerves that run along the testicular artery. These nerves were carefully freed from the surrounding tissue and vasculature, and were cut at two locations with small segments of nerves between the cuts removed [18]. For IWAT denervation surgery, the nerves connecting IWAT with abdominal wall or skin were dissected while keeping the major blood vessels leading into or through IWAT intact [18]. For RWAT denervation surgery, skin incisions on the ventral surface close to the midline and incision into the peritoneal cavity were made to expose the area medial to the kidneys to visualize RWAT [20]. The kidney was gently moved and the nerve bundles that innervate RWAT were exposed and cut [19]. For sham surgeries at intact adipose depots, the nerves of adipose contralateral to the denervated adipose were visualized without being damaged. After surgeries, the denervated and intact adipose tissues were placed back in their original locations. For BAT and IWAT surgeries, the skin incision was closed with wound clips. For EWAT and RWAT surgeries, the peritoneum was sutured with sterile absorbable vicryl sutures and the skin incision was closed with wound clips.

### 2.4. Cannula Placement Verification and icv Injection Procedure

Cannula placement was verified after postoperative recovery by monitoring water drinking following injection of 10 ng angiotensin II (Sigma-Aldrich, St Louis, MO, USA) in 1 μL 0.9% saline in water-replete rats. Briefly, rats received icv injections of 1 μL angiotensin II solution using an internal injector (Plastics One) placed inside the implanted guide cannula. Only those rats that drank more than 5 mL of water within 1 h of angiotensin II injection were included in the subsequent experiments. Body weights and feeding were monitored for one week before the rats receiving saline or BDNF injection which was carried out similarly to the angiotensin II injection.

### 2.5. Body Weight, Body Composition, and Food Intake

Body weight and food were measured before and 24 h after icv saline or BDNF injections. Food intake was calculated by subtracting the weights of food after 24 h from the starting weights and correcting for spillage. Body composition, i.e., fat mass and lean mass, of conscious rats was assessed using an EchoMRI whole-body composition analyzer (EchoMedical Systems, Houston, TX, USA) before and 24 h after icv saline or BDNF injections in the first cohort of rats.

### 2.6. Plasma Lipolytic Products, Corticosterone, and Catecholamines

Plasma samples from the first cohort of rats that received icv saline or BDNF injections without denervation surgeries were measured for lipolytic products, corticosterone, and catecholamines, due to the likelihood that denervation surgeries might impact metabolism or stress hormone levels. Free fatty acids (NEFA-HR2 kit, Wako Chemicals, Richmond, VA, USA), glycerol (free glycerol reagent and glycerol standard solution, Sigma-Aldrich), and corticosterone (Enzo Life Sciences, Farmingdale, NY, USA) were measured using enzymatic colorimetric methods according to the manufacturers’ directions. Plasma epinephrine and NE concentrations were measured using high-pressure liquid chromatography (HPLC).

### 2.7. Catecholamine Extraction and Measurement

Plasma and tissue catecholamine content was measured using a reverse-phase HPLC system (Thermo UltiMate^TM^ 3000 UHPLC, Thermo Fisher Scientific, Waltham, MA, USA) with electrochemical detection (ECD-3000RS, Thermo Fisher Scientific), following an established protocol [15,21] modified from the method of Mefford [22]. Briefly, 100 µL of plasma or ~100 mg of tissue was homogenized in 0.2 M perchloric acid with 1 mg/ml ascorbic acid solution containing the internal standard dihydroxybenzylamine (DHBA, Thermo Fisher Scientific) to control extraction efficiency. After centrifugation, catecholamines were extracted from the homogenate with alumina and 0.5 M tris(hydroxymethyl)aminomethane buffer (pH 8.6) and were eluted into the perchloric acid with ascorbic acid solution. The extracted catecholamines were assayed with Cat-A-Phase II mobile phase (Thermo Fisher Scientific) and separated with a HR-80 reverse phase column (Thermo Fisher Scientific). The electrochemical detector settings were: guard cell, +250 mV; cell 1, +100 mV; and cell 2, −200 mV. Catecholamine values were calculated based on the standard curve from each assay and were corrected for extraction efficiency based on the internal standard DHBA within each sample.

### 2.8. Determination of NETO

NETO was determined using the αMPT method as described previously [21,23]. αMPT is a competitive inhibitor of tyrosine hydroxylase, the rate-limiting enzyme in catecholamine biosynthesis that blocks de novo catecholamine synthesis [24]. αMPT methyl ester hydrochloride (Sigma-Aldrich) was prepared by first adding 1 μL glacial acetic acid/mg αMPT and then diluting to a final concentration of 25 mg/mL with 0.9% sterile saline. After αMPT injection, endogenous NE contents of tissues decline at a rate proportional to NE release from innervation [15].

The effect of BDNF on NETO was assessed according to the established method [15] and a published study that examined the effect of BDNF on adipose tissue NETO [8]. Rats from the second cohort received icv injection of either saline or BDNF. Two hours later [8], half of the saline- and BDNF-treated rats received ip injections of αMPT (250mg/kg, Sigma-Aldrich). This 2 h regimen has also been used to study effects of icv leptin injection on NETO in various adipose tissues [25]. The other half of the rats were given vehicle injection (no αMPT) to provide the baseline NE content needed to calculate NETO. After an additional 2 hours, αMPT-injected rats received a supplemental dose of αMPT (125 mg/kg) to ensure continuous inhibition of tyrosine hydroxylase, and vehicle-injected rats received a second ip vehicle injection. Four hours after initial αMPT or vehicle ip injection, or six hours after icv BDNF or saline injection, the timepoint when BAT thermogenesis is activated [6] and increased BAT *Ucp1* gene expression has been detected [6], rats were euthanized, and intact and denervated BAT, EWAT, IWAT, and RWAT were collected, frozen, and stored at −80 °C until catecholamine extraction. NE content was measured using HPLC, and measurements were used to determine NETO. NETO was calculated by subtracting depleted NE content of αMPT group ([NE]_4_) from baseline NE content of no αMPT group ([NE]_0_). Constant rate of NE efflux (*k*) was calculated using *k* = (lg[NE]_0_ − lg[NE]_4_)/(0.434 × 4). NETO (K) was calculated using K = *k*[NE]_0_ [24].

### 2.9. Analysis of Gene Expression Using Real-Time Quantitative PCR

Genes of interest were analyzed by relative quantitation standardized to the constitutively expressed ribosomal protein L32 (*Rpl32*), using real-time quantitative PCR in intact and denervated adipose tissue of saline- or BDNF-treated rats. Genes of interested included sympathetically regulated genes, *Adrb3* coding β3-AR and *Ucp1* coding UCP1; genes involved in lipolysis, such as *Lipe* coding hormone-sensitive lipase (HSL) and *Pnpla2* coding adipose triglyceride lipase (ATGL); genes related to fatty acid biosynthesis of lipogenesis, such as *Fasn* coding fatty acid synthase and *Acaca* coding acetyl-CoA carboxylase α; and genes related to triglyceride biosynthesis of lipogenesis, such as *Gpam* coding glycerol-3-phosphate acyltransferase and *Dgat1* coding diacylglycerol acyltransferases 1. Detailed procedures are included in the Supplementary Material, as well as an analysis of gene expression. The primer sequences for these *rattus norvegicus* genes are included in Supplementary Material Table S1. The specificities of all the primers were confirmed by sequencing amplified products using conventional Sanger sequencing [26], which were mapped to NCBI Blast with at least 88% match (Supplementary Material Table S2).

### 2.10. Analysis of Protein Expression Using Western Blot

Proteins were extracted using RIPA lysis buffer system (Santa Cruz Biotechnology, Dallas, TX, USA) and loaded onto a 4–20% Tris-HCl gradient gel (Bio-Rad Laboratories). Matched intact and denervated adipose tissues from the same rats were measured in the same gels for side-by-side comparison. Proteins were transferred to nitrocellulose membranes (Bio-Rad Laboratories), which were incubated with one of the following rabbit antibodies (1:1000 dilution) in 5% bovine serum albumin overnight: GAPDH (37 kDa; Cell Signaling Technology, Danvers, MA, USA), phosphorylated HSL (p-HSL; 83 kDa; Cell Signaling Technology), HSL (83 kDa; Cell Signaling Technology), ATGL (54 kDa; Cell Signaling Technology), fatty acid binding protein 4 (FABP4; 15 kDa; Cell Signaling Technology), or UCP1 (33 kDa; Abcam, Cambridge, MA, USA).

The immunoblots were then incubated with 1:5000 horseradish peroxidase-conjugated goat anti-rabbit antibody (Cell Signaling Technology) for 1 h. Detection was achieved using the chemiluminescence system (Amersham™ ECL™ Prime, GE Healthcare, Chicago, IL, USA). A C-Digit blot scanner (LI-COR Biosciences, Lincoln, NE, USA) was used for development and visualization of the proteins, and an Odyssey infrared imaging system was used for protein quantification. For immunoblots, all protein concentrations were normalized to GAPDH on the same membrane. Because p-HSL and HSL have the same protein size, they were assayed on separate gels, and p-HSL/HSL ratio was calculated by normalizing p-HSL/GAPDH to HSL/GAPDH.

### 2.11. Statistical Analysis

All data were presented as mean ± SEM and were analyzed using Prism Statistical Software 8 (GraphPad Prism, La Jolla, CA, USA). Food intake, changes in body weights and body composition, and plasma parameters between icv saline and BDNF treatment groups from the first cohort were compared using unpaired two-tailed t-tests. NE concentration and NETO of intact adipose tissues of icv saline-treated rats from the second cohort were compared using a one-way analysis of variance (ANOVA) followed by Tukey’s post hoc tests. NE concentration, NETO, protein and gene expression of the same type intact and denervated adipose tissues from the second cohort were compared using a two-way ANOVA with innervation and icv treatment as independent variables, followed by Tukey’s post hoc tests. Differences for all tests were considered statistically significant if *p* < 0.05.

## 3. Results

### 3.1. Cohort 1: Effects of BDNF on Whole-Animal Lipid Mobilization

#### 3.1.1. Food Intake, Body Weight, and Body Composition

**Figure 1:**
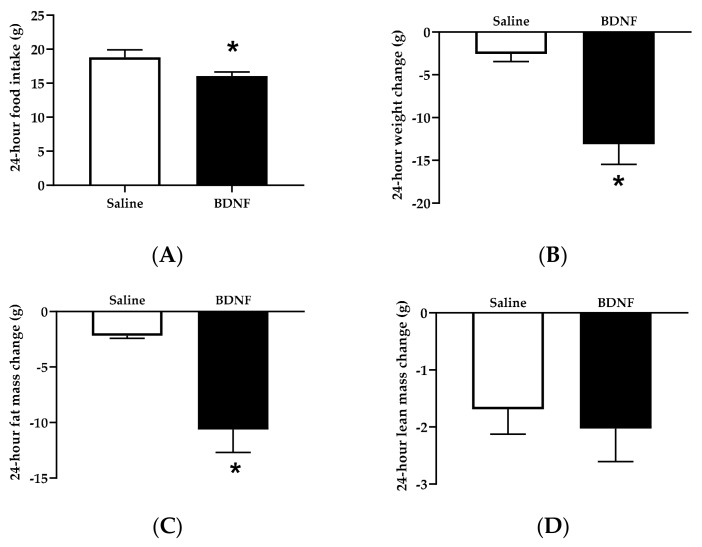
Food intake, body weight change, and body composition change in saline- or BDNF-treated rats. (**A**) Food intake, (**B**) body weight change, and body composition changes in (**C**) fat mass and (**D**) lean mass measured before and 24 hours after intracerebroventricular injections of saline or BDNF. Data are expressed as mean ± SEM, *n* = 10 per group, and unpaired two-tailed t-tests were used to compare between saline and BDNF treatment groups. * Represents significant differences relative to saline-treated rats (*p* < 0.05).

Food intake during the 24 h following icv injection was significantly lower in BDNF-treated rats than saline-treated rats by 14.56 ± 3.12% (t = 2.189, *p* < 0.05; Figure 1A). When 24 h body weight changes were examined, BDNF-treated rats had 4.08 ± 0.91-fold decrease in body weight compared to saline-treated rats (t = 4.173, *p* < 0.001; Figure 1B). Body weight is determined by the combination of fat mass and lean mass. Body composition analysis revealed that BDNF-treated rats had 3.54 ± 0.86-fold reduction in fat mass compared to saline-treated rats (t = 4.069, *p* < 0.001; Figure 1C). In contrast, the average changed amount of lean tissue mass was comparable between saline and BDNF treatment groups (Figure 1D). Therefore, ~15% feeding suppression by icv BDNF administration led to ~ 4-fold weight loss and fat loss.

#### 3.1.2. Circulating Factors

Plasma concentrations of glycerol (t = 2.237, *p* < 0.05) and free fatty acids (t = 3.049, *p* < 0.01) of BDNF-treated rats were significantly higher than those of saline-treated rats, indicating greater circulating levels of lipolytic products in BDNF-treated rats. Plasma levels of corticosterone, epinephrine, and NE were not significantly different between saline- and BDNF-treated groups (*p* > 0.05; Table 1).

#### 3.1.3. Expression of Lipolytic Proteins in Adipose Tissues

**Figure 2:**
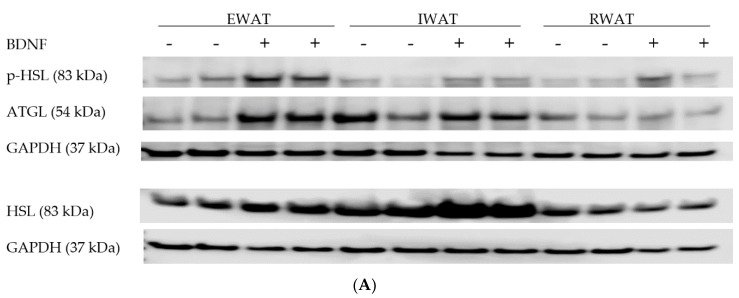

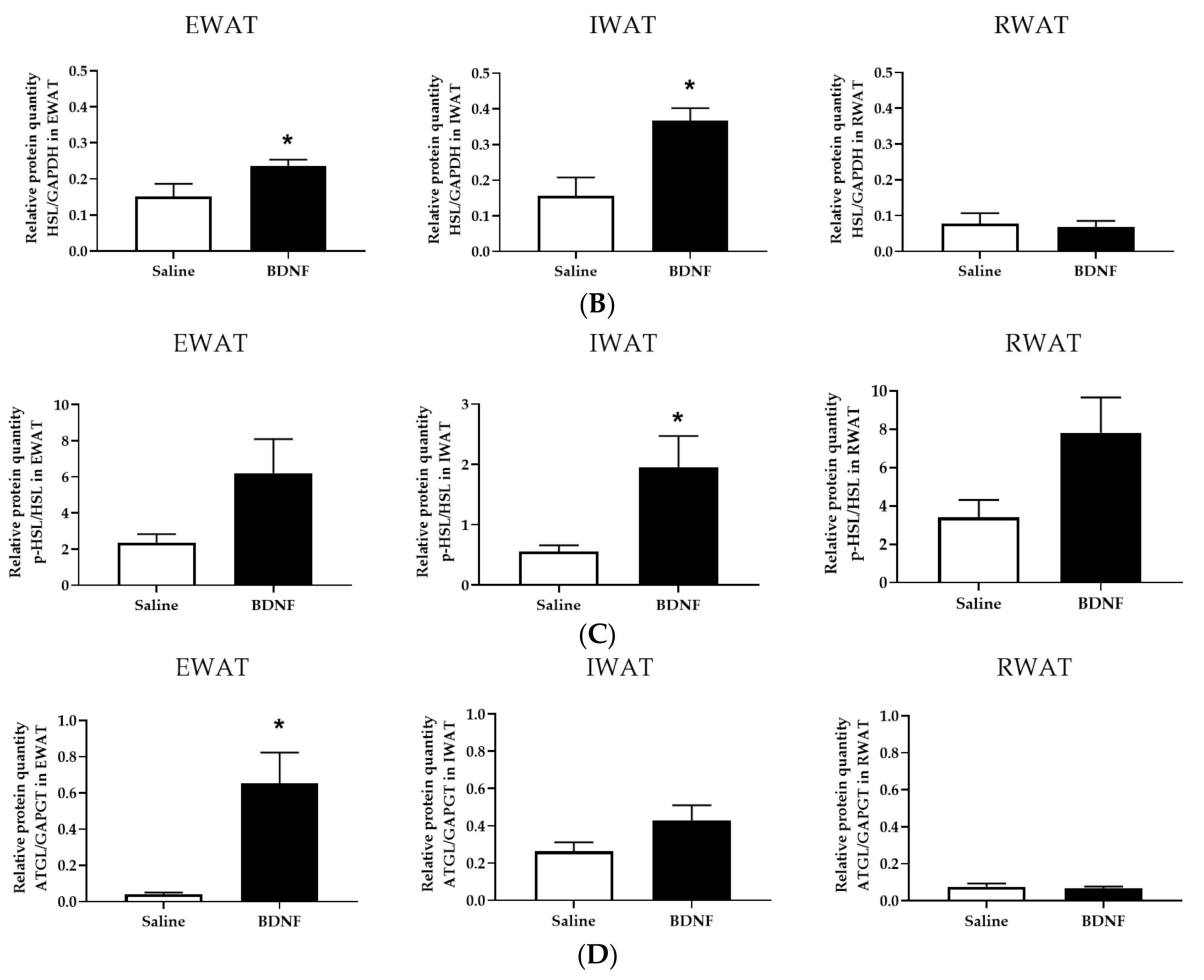
Expression of lipolytic proteins involved in lipid mobilization in white adipose tissues of intracerebroventricular (icv) saline- or BDNF-treated rats. Quantifications of HSL and ATGL were normalized to GAPDH. Quantifications of p-HSL/GAPDH were normalized to HSL/GAPDH to obtain p-HSL/HSL. (**A**) Representative western blot images of expression of p-HSL, HSL, ATGL, and GAPDH in EWAT, IWAT, and RWAT of saline- (−) or BDNF-treated (+) rats. Quantification of (**B**) HSL/GAPDH, (**C**) p-HSL/HSL, and (**D**) ATGL/GAPDH in epididymal white adipose tissue (EWAT), inguinal WAT (IWAT), and retroperitoneal WAT (RWAT) of saline- or BDNF-treated rats. Each sample was loaded on two separate gels, one being labeled with p-HSL, ATGL and GAPDH and the other being labeled with HSL and GAPDH. Data are expressed as mean ± SEM, *n* = 10 per group, and unpaired two-tailed t-tests were used to compare between saline and BDNF treatment groups. * Represents significant differences relative to saline-treated rats (*p* < 0.05).

Because greater circulating concentrations of lipolytic products indicate enhanced lipolysis in BDNF-treated rats, expression of lipolytic proteins p-HSL, HSL, and ATGL were measured in EWAT, IWAT, and RWAT. When normalized to GAPDH, BDNF-treated rats had significantly greater ratios of HSL/GAPDH (Figure 2A,B) in EWAT (t = 2.166, *p* < 0.05) and IWAT (t = 3.329, *p* < 0.01) than saline-treated rats. When p-HSL was normalized to HSL, BDNF-treated rats had significantly greater ratio of p-HSL/HSL in IWAT (t = 2.703, *p* < 0.05) compared to saline-treated rats; whereas p-HSL/HSL ratios in EWAT (t = 1.977, *p* = 0.0549) and RWAT (t = 1.928, *p* = 0.0590) of BDNF-treated rats tended to be greater than those of saline-treated rats (Figure 2A,C). Additionally, the ratio of ATGL/GAPDH in EWAT (t = 2.997, *p* < 0.01), but not IWAT or RWAT, was significantly greater in BDNF-treated than saline-treated rats (Figure 2A,D). These results indicated that acutely administered BDNF enhanced expression of lipolytic enzymes catalyzing lipolysis of EWAT and IWAT.

### 3.2. Cohort 2: Effects of BDNF on Adipose Lipolysis via Sympathetic Activation

#### 3.2.1. Adipose Tissue NE Concentration

Concentration of the sympathetic neurotransmitter NE was very different between WAT and BAT, but was not significantly different among various types of WAT (Figure S1A). When NE concentration was compared between intact and denervated same type adipose tissues, except for denervated BAT (Figure S1B), NE concentration was significantly reduced by denervation (Figure S1C–E). Central injection of BDNF did not affect NE concentration of any intact or denervated adipose tissues (Figure S1). Detailed results were included in the Supplementary Material, adipose tissue NE concentration.

#### 3.2.2. Adipose Tissue NETO

**Figure 3:**
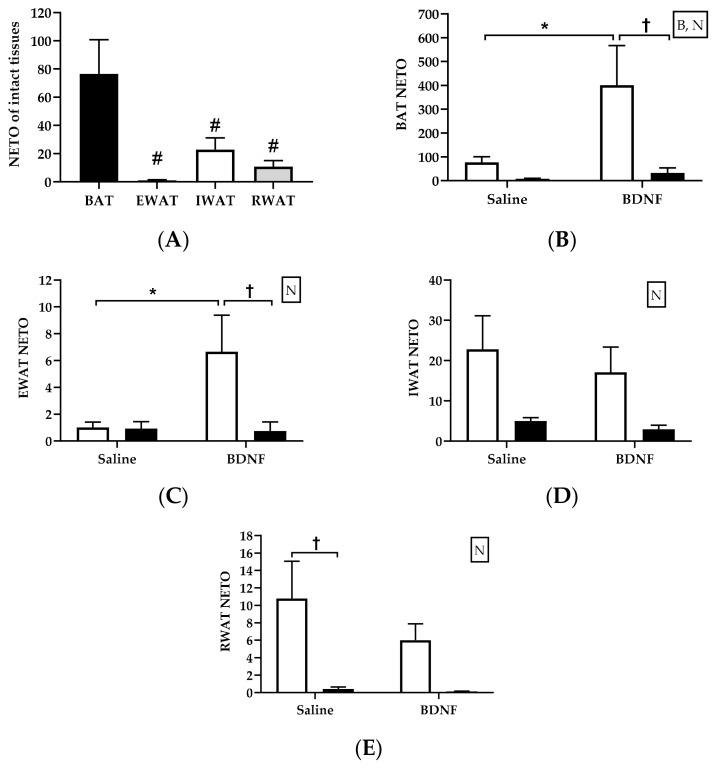
Norepinephrine turnover in intact and denervated brown and white adipose tissues of intracerebroventricular (icv) saline- or BDNF-treated rats. (**A**) Norepinephrine turnover (NETO) in intact brown adipose tissues (BAT), epididymal white adipose tissue (EWAT), inguinal WAT (IWAT), and retroperitoneal WAT (RWAT) of icv saline-treated rats that received intraperitoneal injection of vehicle. One-way analysis of variance (ANOVA) was used to compare NETO of intact adipose tissues in saline-treated rats. (**B**–**E**) NETO in intact (open bar) and denervated (filled bar) BAT, EWAT, IWAT, and RWAT of icv saline- or BDNF-treated rats. Two-way ANOVA with factors “innervation” (N; intact vs. denervated) and “BDNF” (B; icv saline vs. BDNF) and multiple comparisons post hoc Tukey’s test were used to compare NETO. # Represents significant differences relative to BAT (*p* < 0.05). * Represents significant differences relative to saline-treated rats (*p* < 0.05). † Represents significant differences relative to denervated adipose tissue (*p* < 0.05).

Similar to NE concentration, NETO of intact BAT was significantly greater than any of the intact WAT analyzed (F = 6.722, *p* < 0.01; Figure 3A). Multiple comparison tests revealed that, among intact adipose tissues of saline-treated rats, NETO was significantly different between BAT and EWAT (*p* < 0.01), BAT and IWAT (*p* < 0.05), and BAT and RWAT (*p* < 0.01); whereas NETO was not significantly different among WAT depots from various locations (Figure 3A).

Central injection of BDNF differentially affected NETO across various adipose tissues assayed in this study. Specifically, multiple comparison tests revealed that BDNF treatment significantly increased NETO in the intact BAT (*p* < 0.05; Figure 3B) and intact EWAT (*p* < 0.05; Figure 3C); which was abolished in denervated BAT (Figure 3B) and EWAT (Figure 3C). In contrast, NETO in RWAT (Figure 3D) or IWAT (Figure 3E) was not significantly changed by BDNF treatment. Additionally, when NETO was compared between intact and denervated same type adipose tissues, NETO of denervated BAT (F = 6.64, *p* < 0.05; Figure 3B), EWAT (F = 4.31, *p* < 0.05; Figure 3C), IWAT (F = 9.29, *p* < 0.01; Figure 3D), and RWAT (F = 12.07, *p* < 0.01; Figure 3E) were all significantly lower than their respective intact adipose tissues.

#### 3.2.3. Adipose Tissue Gene Expression

Expression of genes related to sympathetic regulation of *Adrb3* (Figure S2A) and *Ucp1* (Figure S2B) in WAT and BAT was not different between saline- and BDNF-treated rats, but was significantly different between intact and denervated adipose tissues at some locations. Expression of *Lipe* gene coding lipolytic enzyme HSL was significantly suppressed by denervation in BAT (Figure S2C). Additionally, expression of *Pnpla2* gene coding lipolytic enzyme ATGL was significantly suppressed by denervation in BAT and EWAT, but not in IWAT or RWAT (Figure S2D). Expression of lipogenic genes involved in fatty acid synthesis (*Acaca* and *Fasn*; Figure S2E,F) and triglyceride synthesis (*Gpam* and *Dgat1*; Figure S2G,H) was also lower in denervated BAT and EWAT, but not in RWAT or IWAT, compared to their respective intact adipose tissues. Additionally, BDNF treatment significantly reduced expression of *Gpam* in RWAT (Figure S2G). Detailed results were included in the Supplementary Material, adipose tissue gene expression.

#### 3.2.4. Adipose Tissue Protein Expression

**Figure 4:**
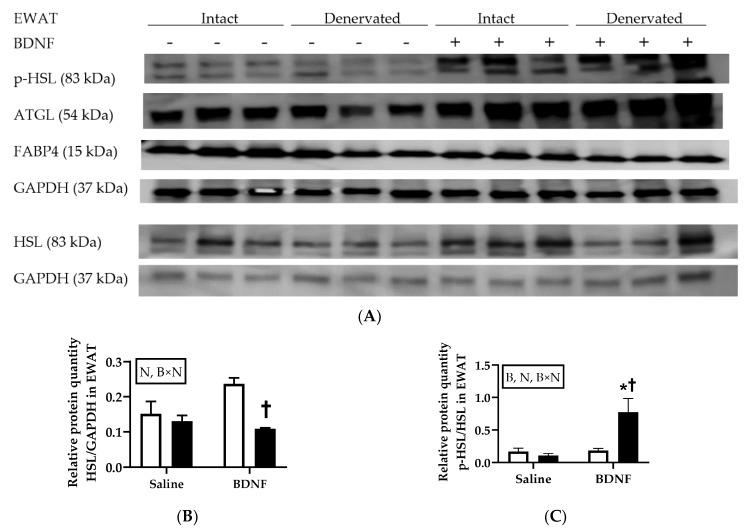

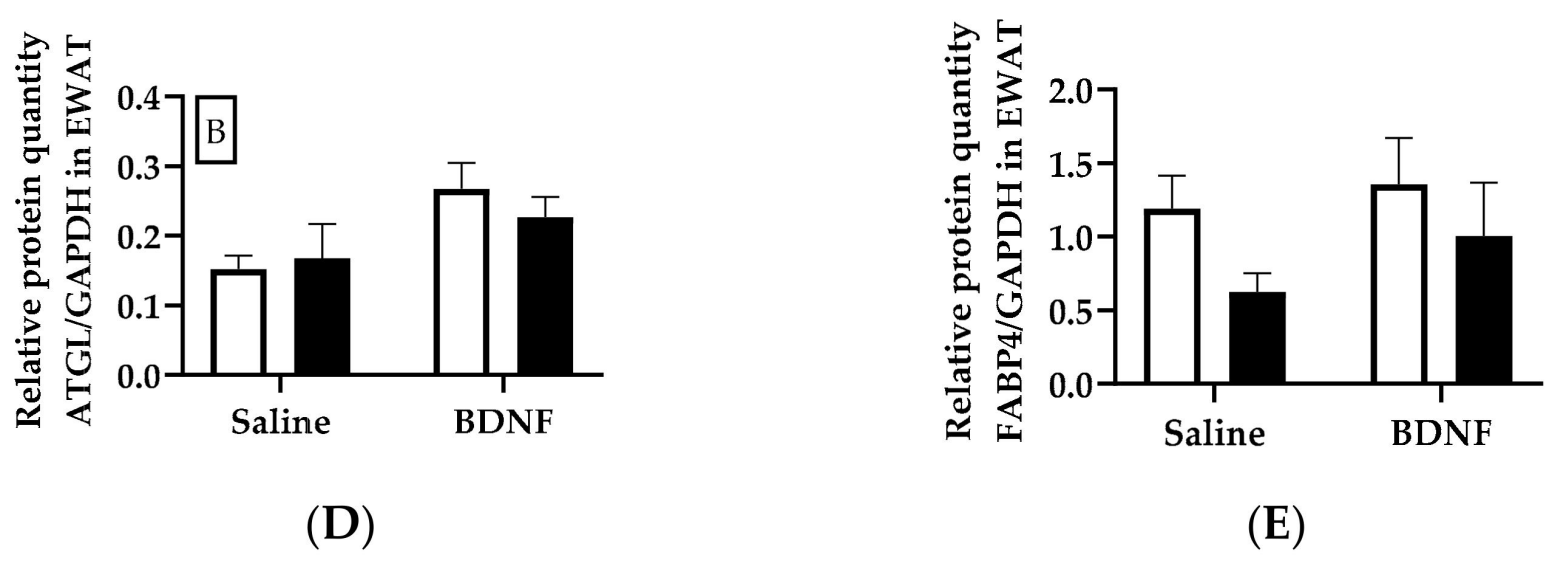
Expression of lipolytic p-HSL, HSL, ATGL, and FABP4 in intact and denervated epididymal white adipose tissue (EWAT) of intracerebroventricular saline- or BDNF-treated rats. Quantifications of p-HSL, HSL, ATGL, and FABP4 were normalized to GAPDH. Quantifications of p-HSL/GAPDH were also normalized to HSL/GAPDH to obtain p-HSL/HSL. (**A**) Representative western blot images of expression of p-HSL, HSL, ATGL, and GAPDH; and quantifications of (**B**) HSL/GAPDH, (**C**) p-HSL/HSL, (**D**) ATGL/GAPDH, and (**E**) FABP4/GAPDH in intact (open bar) and denervated (filled bar) EWAT of saline- or BDNF-treated rats. Two-way ANOVA with the factors “innervation” (N; intact vs. denervated) and “BDNF” (B; icv saline vs. BDNF treatment) and multiple comparisons post hoc Tukey’s test were used to compare relative protein quantities. * Represent significant differences relative to saline-treated rats (*p* < 0.05). † Represent significant differences relative to denervated adipose tissue (*p* < 0.05).

When normalized to an internal control protein GAPDH, the HSL/GAPDH ratio in EWAT was significantly decreased by denervation (F = 12.20, *p* < 0.01) and interaction of denervation and BDNF treatment (F = 6.28, *p* < 0.05). Multiple comparison test revealed significant differences in the HSL/GAPDH ratio between intact and denervated EWAT of BDNF-treated rats (*p* < 0.05; Figure 4A,B). When p-HSL was normalized to HSL, p-HSL/HSL ratio in EWAT was affected by denervation (F = 5.56, *p* < 0.05), BDNF treatment (F = 9.35, *p* < 0.05), and interaction of denervation and BDNF treatment (F = 8.44, *p* < 0.05), with denervated EWAT of BDNF-treated rats having greater p-HSL/HSL ratio than denervated EWAT of saline-treated rats (*p* < 0.05) and intact EWAT of BDNF-treated rats (*p* < 0.05; Figure 4A,C). Two-way ANOVA revealed that ATGL/GAPDH ratio in EWAT was significantly increased by BDNF treatment (F = 6.06, *p* < 0.05), although post hoc multiple comparison test did not reveal significant difference between any treatments (Figure 4A,D). Neither BDNF treatment nor denervation significantly affected the expression of FABP4 protein in EWAT (Figure 4A,E).

**Figure 5:**
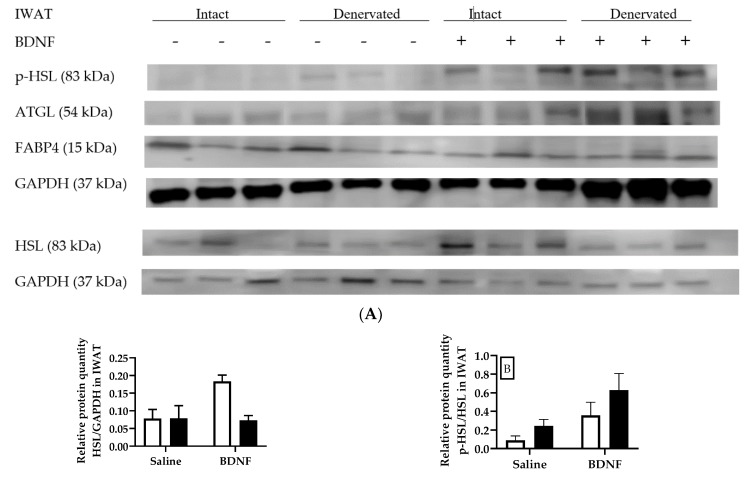

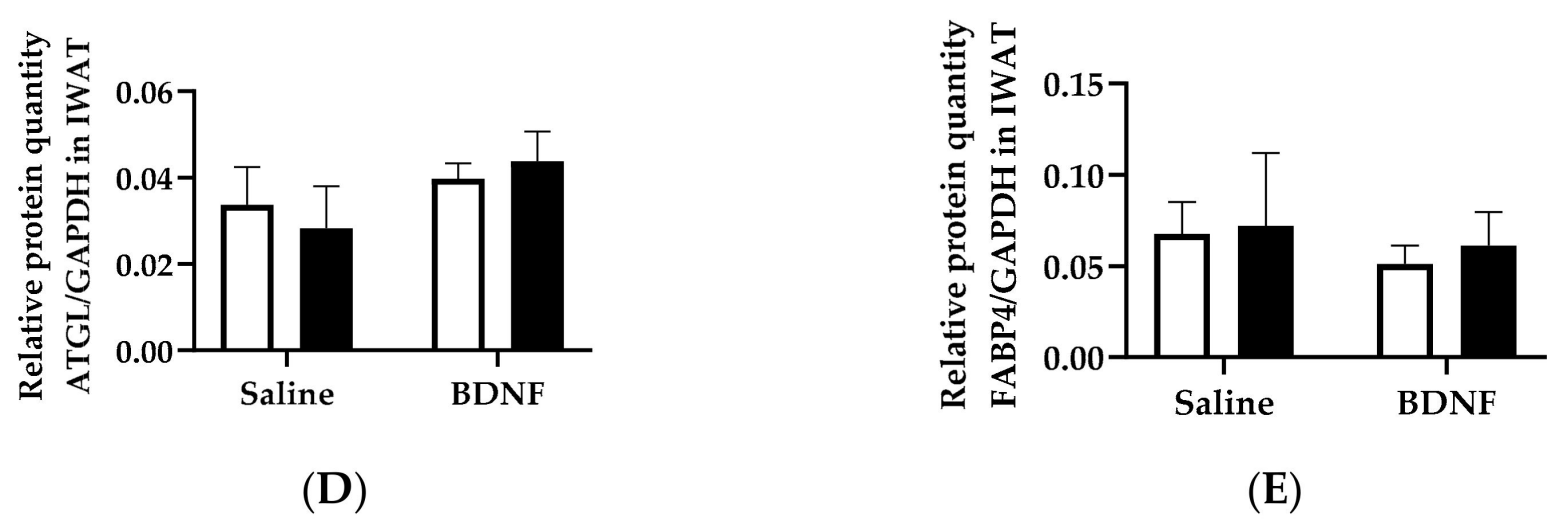
Expression of lipolytic p-HSL, HSL, ATGL, and FABP4 in intact and denervated inguinal white adipose tissue (IWAT) of intracerebroventricular saline- or BDNF-treated rats. Quantifications of p-HSL, HSL, ATGL, and FABP4 were normalized to GAPDH. Quantifications of p-HSL/GAPDH were also normalized to HSL/GAPDH to obtain p-HSL/HSL. (**A**) Representative western blot images of expression of p-HSL, HSL, ATGL, and GAPDH; and quantifications of (**B**) HSL/GAPDH, (**C**) p-HSL/HSL, (**D**) ATGL/GAPDH, and (**E**) FABP4/GAPDH in intact (open bar) and denervated (filled bar) IWAT of saline- or BDNF-treated rats. Two-way ANOVA with the factors “innervation” (N; intact vs. denervated) and “BDNF” (B; icv saline vs. BDNF treatment) and multiple comparisons post hoc Tukey’s test were used to compare relative protein quantities. * Represents significant differences relative to saline-treated rats (*p* < 0.05).

In IWAT, BDNF treatment tended to increase the HSL/GAPDH ratio (F = 4.02, *p* = 0.0799), whereas denervation tended to decrease it (F = 4.83, *p* = 0.0592). Multiple comparison post hoc test revealed that, compared with saline treatment, BDNF treatment tended to increase the HSL/GAPDH ratio in intact IWAT (*p* = 0.0669), but not in denervated IWAT (Figure 5A,B). Additionally, the HSL/GAPDH ratio tended to be lower in denervated IWAT than in intact IWAT of BDNF-treated rats (*p* = 0.0552; Figure 5B). The p-HSL/HSL ratio in IWAT was significantly increased by BDNF treatment (F = 7.24, *p* < 0.05), although multiple comparison did not reveal any significant difference between any treatments (Figure 5A,C). Neither BDNF treatment nor denervation significantly affected protein expression of ATGL or FABP4 in IWAT (Figure 5A,D,E).

**Figure 6:**
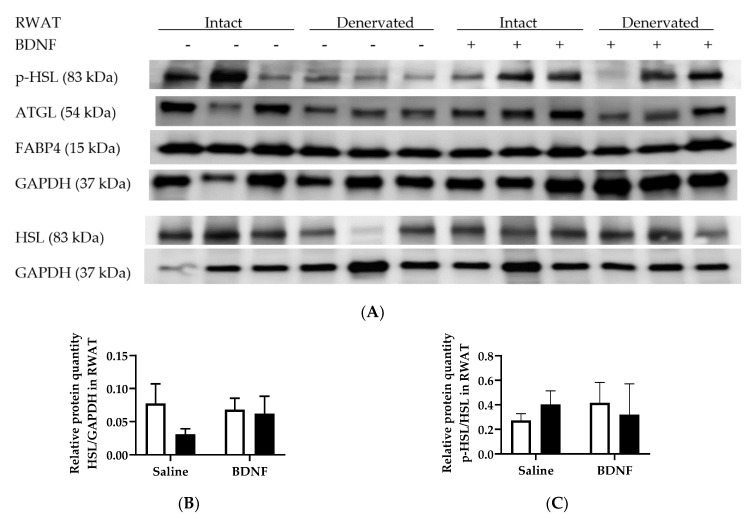

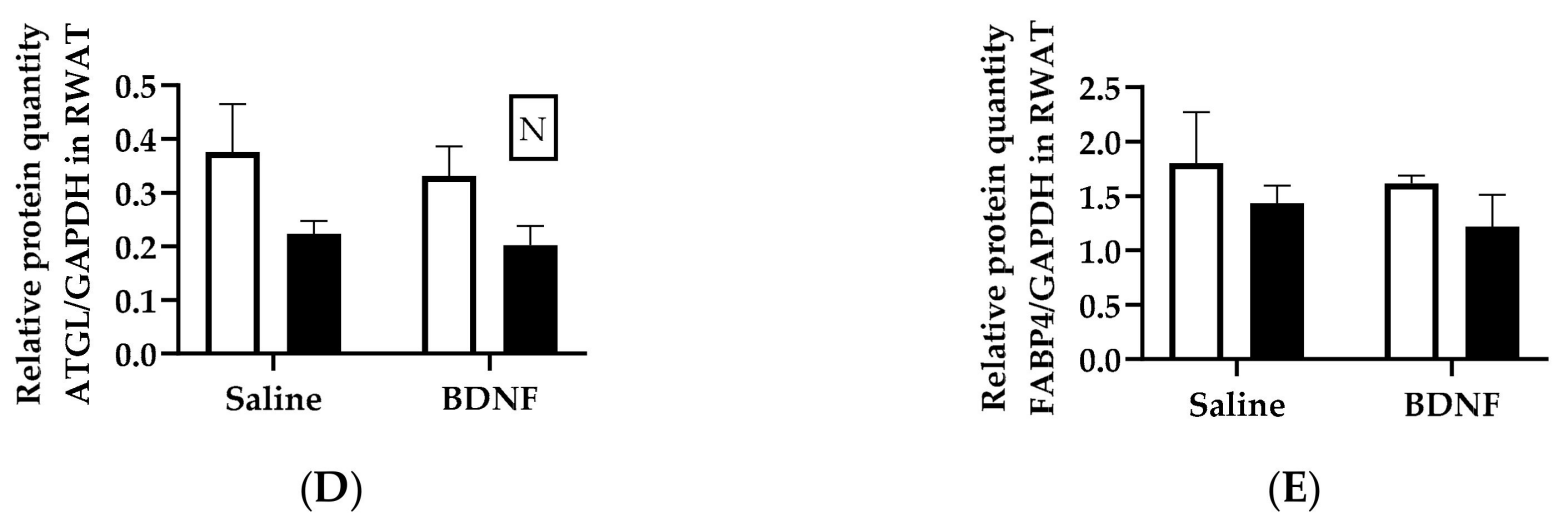
Expression of lipolytic p-HSL, HSL, ATGL, and FABP4 in intact and denervated retroperitoneal white adipose tissue (RWAT) of intracerebroventricular saline- or BDNF-treated rats. Quantifications of p-HSL, HSL, ATGL, and FABP4 were normalized to GAPDH. Quantifications of p-HSL/GAPDH were also normalized to HSL/GAPDH to obtain p-HSL/HSL. (**A**) Representative western blot images of expression of p-HSL, HSL, ATGL, and GAPDH; and quantifications of (**B**) HSL/GAPDH, (**C**) p-HSL/HSL, (**D**) ATGL/GAPDH, and (**E**) FABP4/GAPDH in intact (open bar) and denervated (filled bar) RWAT of saline- or BDNF-treated rats. Two-way ANOVA with the factors “innervation” (N; intact vs. denervated) and “BDNF” (B; icv saline vs. BDNF treatment) and multiple comparisons *post hoc* Tukey’s test were used to compare relative protein quantities.

The ATGL/GAPDH ratio in RWAT was significantly decreased by denervation (F = 6.07, *p* < 0.05), although multiple comparison did not reveal significant difference between any treatments (Figure 6A,D). HSL/GAPDH, p-HSL/HSL, or FABP4/GAPDH in RWAT was not significantly different among any group (Figure 6B,C,E).

**Figure 7:**
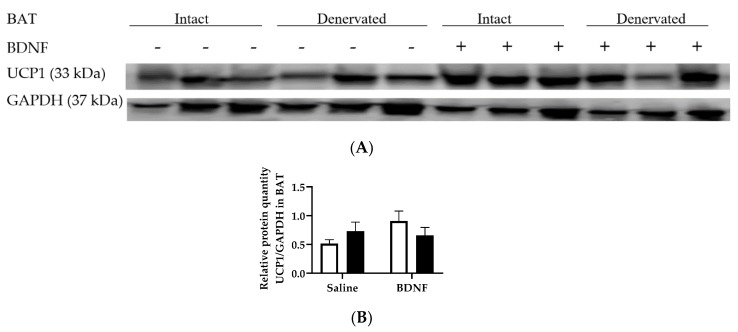
Expression of UCP1 in intact and denervated interscapular brown adipose tissues (BAT) of intracerebroventricular saline- or BDNF-treated rats. Quantification of UCP1 was normalized to GAPDH. (**A**) Representative western blot image of expression of UCP1 and GAPDH; and (**B**) quantification of UCP1/GAPDH in intact (open bar) and denervated (filled bar) BAT of saline- or BDNF-treated rats. Two-way ANOVA was used with the factors “innervation” (N; intact vs. denervated) and “BDNF” (B; icv saline vs. BDNF treatment) and multiple comparisons *post hoc* Tukey’s test were used to compare relative protein quantities.

UCP1 protein expression in BAT was not significantly changed by either BDNF treatment or denervation (Figure 7A,B).

## 4. Discussion

### 4.1. Summary of Findings

Findings from the first cohort demonstrated enhanced lipid mobilization by central administration of BDNF. Specifically, adipose mass decreased following BDNF treatment, beyond what could be accounted for by BDNF-induced inhibition in feeding, indicating enhanced lipid mobilization by BDNF. In addition, circulating concentrations of lipolytic products glycerol and free fatty acids, but not circulating levels of corticosterone, epinephrine, or NE, were significantly higher in the BDNF-treated group compared to the saline-treated group, indicating that BDNF stimulated lipolysis without increasing levels of adrenal medullary catecholamines or cortical glucocorticoid. This finding suggested that BDNF-induced lipid mobilization was via sympathetic innervation but not via activation of the adrenocortical or adrenomedullary system. Circulating concentrations of lipolytic products are indicators of a global lipid mobilization in whole animals. Indeed, further analysis indicated that expression of lipolytic enzymes in major visceral EWAT and subcutaneous IWAT were greater in BDNF-treated than saline-treated rats. These results further supported that central BDNF administration increased lipid mobilization via triggering adipose lipolysis.

BDNF stimulates adipose lipolysis, perhaps via the activation of BDNF- and/or TrkB-expressing neurons located in sympathetic outflow circuits from the brain that ultimately innervate WAT and BAT [13]. This sympathetic activation typically correlates with increases in circulating concentrations of free fatty acids and glycerol and decreased body fat mass [1]. Findings from the first cohort showing that plasma concentrations of adrenal cortical corticosterone and medullary catecholamines were not increased following BDNF administration provide further support for this possible sympathetic nerve-mediated mechanism underlying BDNF-triggered increases in plasma concentrations of fatty acids and glycerol. A few in vivo studies have examined the effects of BDNF on the activity of sympathetic nerves in BAT [8,12], but to our knowledge, a comparison of the effects of central BDNF administration on sympathetic activity among individual adipose depots at different locations has not been reported. In the second cohort of rats with unilateral denervation, we found that icv injection of BDNF did not cause a uniform NETO response among the assessed adipose depots. Specifically, selective sympathetic activation in EWAT and BAT with intact innervation occurred following central BDNF administration. It is noteworthy that sympathetic activity, indicated by NETO, was lower in all denervated BAT and WAT than their respective intact tissues, indicating successful denervation. NE concentration at baseline ([NE]_0_), in contrast, was significantly lower in all denervated WAT, but not BAT, possibly due to circulating NE arising from the adrenal gland making up the majority of total NE that reaches BAT via its rich blood supply [27]. Although NE concentrations in EWAT and BAT were not affected by BDNF treatment, the NETO in these two adipose tissues was greatly enhanced by BDNF treatment. These results clearly demonstrated that BDNF does not affect tissue NE content, but it increases sympathetic drive in intact EWAT and intact BAT. Additionally, sympathetic activation at BAT and EWAT disappeared following denervation of BAT and EWAT, suggesting that BDNF-induced adipose tissue sympathetic activity was dependent on intact innervation, and further supporting the idea that BDNF stimulates adipose tissue lipolysis via direct sympathetic innervation.

### 4.2. Sympathetic Activity Indicated by NETO

Simultaneous measurement of NETO in multiple tissues has been widely used as a measure of site-specific sympathetic activity in various conditions including WAT removal [23], BAT transplantation [21], food deprivation, cold exposure or glucoprivation by 2-deoxy-D-glucose treatment [28,29], in BAT of hibernating rodents under different seasonal photoperiods [30], in rodents with elevated energy expenditure [31], and in rodents centrally treated with leptin [25] or melanocortin agonist [32]. Each condition has been reported to produce unique patterns of differential sympathetic drive across different types of adipose tissues. Selective sympathetic drive to EWAT and BAT, but not to IWAT or RWAT, was provoked by central BDNF in the present study. The finding of enhanced NETO in thermogenic BAT is consistent with published studies in which BDNF enhances sympathetic activity and heightens NETO in BAT of *db/db* mice [8,12].

Mobilization of lipid from major adipose depots is not uniform in response to energetically demanding conditions in rodents and humans [33]. Adipose depot-specific increase in NETO and stimulatory effects on lipolysis promoted by physiological and pharmacological stimuli exhibit a signature pattern of sympathetic activation dependent on adipose tissue type. Indeed, adipose depot-specific responses are almost invariably found if two or more adipose depots are assayed, as has been reported in both in vitro and in vivo studies. For example, in vitro lipolytic responses to adrenergic stimulation varies among adipocytes harvested from various adipose tissues [34,35]; and in vivo disparate decreases in adipose mass and adipocyte size of various WAT, indicative of increased lipid mobilization [36], occur in response to diverse stimuli such as estrogen [37], high-fat diet feeding [38], or fasting [39]. The variability in NETO across different adipose depots observed in this study confirmed the notion of disparate, adipose depot-specific responses.

To further investigate whether intact innervation is required for increased NETO of WAT and BAT induced by BDNF, the rats with unilateral denervation of WAT and BAT in the second cohort received icv injections of saline or BDNF. Unilateral denervation was performed so that intact and contralateral denervated adipose tissues shared similar circulating factors. The results showed that BDNF-induced NETO in intact BAT and EWAT disappeared in the denervated BAT and EWAT (Figure 4). Therefore, increased NETO in BAT and EWAT by BDNF relied on intact innervation. To investigate if increased sympathetic drive affects lipid metabolism at the molecular level, expression of certain genes and proteins related to lipid metabolism and sympathetic regulation in individual WAT and BAT were analyzed following a single icv injection of BDNF.

### 4.3. Expression of Lipolytic Genes and Proteins

A few lipolytic proteins were measured in this study. Lipases including p-HSL, HSL, and ATGL that have been shown to undergo lipolysis regulated by sympathetic activity were studied [1]. Fatty acid transporter protein FABP4 was also measured. FABP4 provides feedback regulation of lipolysis via transporting the lipolytic product free fatty acids out of cells that prevents accumulation of fatty acids inside adipocytes [40]. Thus, lipolysis promoted by FABP4 is not directly regulated by sympathetic activity.

Consistent with enhanced whole-animal lipolysis and the selective sympathetic activation detected 6 hours after icv BDNF injection, increased protein expression of lipases, such as p-HSL in EWAT and IWAT and ATGL in EWAT, was observed in both cohorts at this timepoint. To further investigate whether intact innervation is required for BDNF-induced increase in expression of lipases in WAT, the rats with unilateral denervation of WAT and BAT in the second cohort received icv injections of saline or BDNF. The results showed that BDNF-induced expression of p-HSL persisted in denervated EWAT, and thus was independent of intact innervation; whereas BDNF-induced expression of p-HSL expression in IWAT disappeared in denervated IWAT, and thus was reliant on intact innervation. In contrast to the changed expression of lipases, expression of FABP4, a protein that is not directly regulated by sympathetic activity, was not affected by either denervation or BDNF treatment. These findings further supported the idea that BDNF induces lipolysis via sympathetic innervation.

### 4.4. Potential Mechanisms of BDNF Action via SNS

The exact mechanism underlying divergent sympathetic outflow and consequently differential lipolysis across WAT depots is not precisely known but could be due to several possible neuroanatomical explanations. First, a variation in the degree of innervation and divergent sympathetic outflow circuits from the CNS to different WAT depots have been reported [41,42]. Specifically, relatively distinct preganglionic neurons in the intermediolateral horn of the spinal cord and forebrain of rats [42] and postganglionic neurons in the sympathetic chain ganglia of hamsters [41] innervating different WAT depots have been reported. These separate populations of sympathetic outflow neurons to different WAT depots at preganglionic and postganglionic levels could account for adipose depot-specific differences in NETO and thereby differential rates of lipid mobilization. Second, differences in catecholamine concentrations and differences in affinity, expression and ratio of adrenergic receptors [43,44] have been reported in different adipose tissues, which could also be an important contributor in the differential mobilization of lipid from WAT. Third, WAT appears to be a heterogeneous organ with marked variation in its cellular and molecular contents depending on its anatomical location. For example, mitochondrial content and mitochondrial enzymatic activity are greater in EWAT than in IWAT [45]. Additionally, changes in EWAT mass are mostly due to change in cell volume, as determined by size of lipid droplets and lipolysis, whereas changes of IWAT mass and RWAT mass are mostly due to changes in cell numbers [46]. Thus, EWAT could be more metabolically active, especially in lipolysis, than IWAT and RWAT. These region-specific differences imply that the variable responses of different adipose tissues to endocrine and neural factors at biochemical and molecular levels were not unique to the experiments reported here.

Most changes in adipose gene expression were caused by denervation rather than by BDNF treatment, likely due to the acute treatment nature, i.e., single injection of BDNF. The protein levels of lipolytic enzymes were also measured. Expressions of p-HSL and HSL were selected as intracellular markers of SNS-stimulated lipolysis due to their critical roles in triacylglycerol hydrolysis [47]. Central BDNF administration did not affect *Lipe* mRNA level in either EWAT or IWAT, indicating that there was no significant change in *Lipe* gene transcription that could account for translational changes in HSL synthesis or posttranslational changes in HSL activity. Indeed, western blot analysis revealed significantly increased p-HSL and HSL protein expression in intact EWAT and IWAT by BDNF treatment. Furthermore, ATGL, another important lipase in adipocytes, is also predominantly involved in lipolysis and appears to be required for PKA stimulation of fatty acid release in the absence of HSL [48]. Similar to the gene and protein expression of HSL, ATGL protein level, but not *Pnpla2* mRNA level, in EWAT of BDNF-treated rats was higher compared to saline-treated rats. Therefore, although HSL or ATGL transcription was not changed at this timepoint of 6 hours post BDNF administration, HSL and ATGL protein levels and HSL activation increased. UCP1 is a mitochondrial protein that regulates energy expenditure involving thermogenesis. It has been reported that 6 hours after BDNF is directly injected into the hypothalamic paraventricular nucleus, the level of *Ucp1* mRNA increases in BAT of rats [6]. Although the present study revealed a trend towards increased UCP1 protein expression, it did not indicate significantly increased expression of either *Ucp1* gene or UCP1 protein in BAT of BDNF-treated rats compared to saline-treated rats. Therefore, similar gene expressions but distinct protein expressions in WAT and BAT were detected between saline- and BDNF-treated rats. Such discrepancy between gene expression and protein expression is not uncommon, as transcript and protein levels of the same genes do not absolutely correlate due to regulation of translation and protein turnover [49].

### 4.5. Potential Sites of BDNF Action

There is a widespread expression of BDNF receptor TrkB in the CNS [9]. The exact central action sites reached by the icv-delivered BDNF are unknown and could be anywhere periventricularly from the hypothalamus to the spinal cord, where TrkB receptors are located in the sympathetic outflow from the brain to WAT and BAT. We speculated that injection of BDNF into the third ventricle of the hypothalamus, an area adjacent to several discrete nuclei that express the functional full-length form of TrkB receptors, including the paraventricular, dorsomedial, and arcuate nuclei [9], activated sympathetic neurons located in these hypothalamic nuclei and subsequent order of nuclei in the intermediolateral horn of the spinal cord, initiated sympathetic outflow from the CNS to peripheral tissues, increased sympathetic drive to EWAT and BAT, and produced selective increases in NETO, which required intact innervation of these adipose depots. A recent study has reported over 60% coexpression of BDNF-expressing neurons and neurons that are part of the sympathetic outflow from the brain to BAT at some periventricular sites, such as the paraventricular, dorsomedial, and lateral hypothalamic areas [13]. Therefore, it is plausible that BDNF acts via the SNS and modulates sympathetic nerve activity through central regulation of sympathetic outflow neurons in the hypothalamus to affect energy metabolism. It is noteworthy that some BDNF protein produced in one site of the brain can be carried away by anterograde transport within the nervous system [50,51]. Thus, it is possible that BDNF injected into the third ventricle of the hypothalamus could be transported to extra-hypothalamic areas.

Central administration of BDNF has been used in many published studies. Peripherally delivered BDNF encounters two major obstacles in arriving at the CNS: its low blood-brain barrier penetrability, and its short half-life (on the order of minutes) during circulation in blood [52]. In comparison with subcutaneous administration, a much lower dose (approximately 1/100) of BDNF was found to be effective with central administration [8]. The half-life of BDNF in lumbosacral CSF samples following intrathecal injections of BDNF in rats is 62.7 min [53]. BDNF concentration in the brain is stable for at least one hour after intravenous BDNF infusion in mice [54,55]. It is likely that the half-life of icv BDNF in the brain is on the order of hours [56].

TrkB is expressed in adipose tissues at the highest level among a few peripheral tissues [57]. If centrally injected BDNF reaches the periphery, it could directly affect adipose tissues. The dose of 0.3 µg BDNF used in this study is the minimal dose in terms of its ability to inhibit food intake in male rats [16], and is a lower dose than those used to examine central effects of BDNF in many published studies [6,7,8,58]. Because of the low dose, single injection of 0.3 µg BDNF used in this study, it was unlikely to leak into the periphery, a potential confounder often encountered with centrally administered substances. In addition, the possibility of leakage of BDNF into the periphery would not explain the differential increases in WAT NETO across different locations of WAT. In the future, the direct effects of BDNF on adipocyte lipolysis will be assessed in WAT at different locations following in vivo peripheral administration of BDNF, and will be assessed in cultured ex vivo WAT collected from different depots and treated by BDNF, to confirm exclusion of BDNF peripheral action.

### 4.6. Future Studies

Increased NETO in WAT is assumed to be the principal contributor of increased lipolysis leading to elevated circulating free fatty acids and glycerol. BDNF-induced increase in NETO was observed selectively in EWAT, but not in IWAT or RWAT. Only bilaterally presented WAT depots were analyzed in this study, whereas WAT depots at other locations, such as mesenteric WAT, a lipolytically active WAT and a major contributor to circulating lipolytic products, were not tested. Additionally, although the majority of triglycerides are stored in white adipocytes, other tissues and cells that were not assayed in the current study, such as the liver and muscle, also store triglycerides, which could potentially contribute to the increase in the circulating concentrations of lipolytic products following BDNF treatment. Thus, there is a need for future studies to assay BDNF-induced sympathetic activities and lipolysis of the liver, other WAT, and muscle which also contribute to lipid mobilization. BDNF did not change NETO in IWAT or RWAT, which does not exclude the possibility that BDNF could increase lipid metabolism in IWAT and/or RWAT independent of an increase in SNS drive. One possibility is that central BDNF treatment could trigger increases in other lipolytic hormones, such as glucagon [59]. Further studies investigating hormone changes on BDNF treatment are needed to completely rule out this possibility. Although BDNF increased NETO in EWAT and BAT, we did not determine whether increased sympathetic drive to EWAT and BAT would increase lipolysis, inhibit lipogenesis, and/or enhance BAT thermogenesis. Further studies are needed to identify the metabolic pathways that lead to a reduction in fat mass in rats treated with BDNF, and how the individual adipose depots respond to BDNF.

### 4.7. Conclusions

WAT plays a significant role in energy homeostasis by both storing and releasing energy, while BAT mainly mobilizes energy for thermogenesis. BDNF alters metabolism via regulating the sympathetic drive to both WAT and BAT. As demonstrated in the present study, the combination of increased plasma glycerol and free fatty acid concentrations without changes in plasma corticosterone, epinephrine or NE concentrations indicates BDNF enhanced lipolysis via increasing sympathetic activity of adipose tissues. Findings from this study also provide compelling evidence supporting that BDNF reduces body fat mainly via stimulation of sympathetic drive in a tissue-specific manner to EWAT and BAT, as indicated by significant increases in NETO in these tissues, which was dependent on intact innervation. These experiments demonstrate for the first time that central BDNF treatment stimulates the sympathetic drive to WAT and BAT to increase lipid mobilization in a manner that is not uniform among adipose depots, which adds to the evidence of differential sympathetic drive to peripheral adipose tissues.

## Figures and Tables

**Table 1:** Plasma levels of glycerol, free fatty acids, corticosterone, epinephrine, and norepinephrine following intracerebroventricular injection of saline or brain-derived neurotrophic factor (BDNF). Blood samples were collected 6 hours after treatment. *n* = 10 per group. Values are represented as mean ± SEM. * Represents significant differences relative to saline-treated rats (*p* < 0.05).

Parameter	Saline	BDNF
Glycerol (mg/L)	4.9184 ± 0.6983	8.4047 ± 1.3931 *
Free fatty acids (mmol/L)	0.3381 ± 0.0197	0.4234 ± 0.0198 *
Corticosterone (ng/mL)	496.6319 ± 99.2159	403.2675 ± 116.7263
Norepinephrine (ng/mL)	7.5759 ± 3.1380	9.2376 ± 2.3438
Epinephrine (ng/mL)	51.5562 ± 22.5392	37.9994 ± 9.0009

## Supplementary Materials

The following are available online at https://www.mdpi.com/2218-273X/9/9/452/s1. Supplemental methods and results 1: Analysis of gene expression using real-time quantitative PCR; Supplemental results 2: Adipose tissue norepinephrine concentration; Figure S1: Norepinephrine content in intact and denervated brown and white adipose tissues of intracerebroventricular (icv) saline- or BDNF-treated rats; Figure S2: Expression of genes in intact and denervated brown and white adipose tissues of intracerebroventricular (icv) saline- or BDNF-treated rats; Table S1: Primers for real-time quantitative PCR; Table S2: PCR products sequenced using Sanger sequencing and mapped to NCBI Blast to reveal match, gaps and mismatch.
