# Supplementary material for: Differential Sympathetic Activation of Adipose Tissues by Brain-Derived Neurotrophic Factor
Source: Biomolecules. 2019 Sep 5;9(9):452. doi: 10.3390/biom9090452 (PMC6769916; doi:10.3390/biom9090452)

Supplementary Materials

1. Supplemental Material Adipose Tissue Gene Expression

1.1. Methods: *Analysis of Gene Expression Using Real-time Quantitative PCR*

Total RNA was extracted using RNeasy lipid tissue column kit with DNase digestion during RNA purification (QIAGEN, Valencia, CA) according to the manufacturer's protocol. The concentration and quality of the total RNA were determined by a NanoDrop™ 2000 spectrophotometer (Thermo Fisher Scientific). One microgram of the extracted RNA was used to synthesize first-strand cDNA using an iScript kit (Bio-Rad Laboratories, Hercules, CA, USA).

Sympathetically regulated genes *Adrb3* coding β3-AR and *Ucp1* coding UCP1; genes involved in lipolysis, such as *Lipe* coding hormone-sensitive lipase (HSL) and *Pnpla2* coding adipose triglyceride lipase (ATGL); genes related to fatty acid biosynthesis of lipogenesis, such as *Fasn* coding fatty acid synthase and *Acaca* coding acetyl-CoA carboxylase *α*; and genes related to triglyceride biosynthesis of lipogenesis, such as *Gpam* coding glycerol-3-phosphate acyltransferase and *Dgat1* coding diacylglycerol acyltransferases 1, were analyzed by relative quantitation standardized to the constitutively expressed ribosomal protein L32 (*Rpl32*), and expressed relative to gene expression of the matched same type intact adipose tissue of saline-treated rats. The primer sequences for these *rattus norvegicus* genes are included in supplemental material Table S1. Specificity of all these primers were confirmed by sequencing amplified products separated on agarose gel using conventional Sanger sequencing [1]. Briefly, amplicon from each gene product was purified from the agarose gel and sequenced with the BigDye™ Cycle Sequencing kit (Applied Biosystems, Thermo Fisher Scientific) according to manufacturer's instructions, and run on an ABI Prism 3730xl automatic sequencer (Applied Biosystems). Results were mapped to NCBI Blast with at least 88% match (supplemental material Table S2).

Real-time quantitative PCR reactions were performed as described previously [1] using iQ™ SYBR® Green Supermix (Bio-Rad Laboratories) as the detector fluorophore and a real-time PCR detection system (CFX96TM, Bio-Rad Laboratories). Templates (40 ng of cDNA) for each reaction were run in triplicates, with amplifying conditions at 95 °C for 2 min followed by 40 cycles of amplification at 95 °C for 30 s, and annealing at 55 °C for 30 s. Melt curve analysis was performed to ensure specificity of amplification. Identity of the amplification products was confirmed by gel electrophoresis. Relative mRNA expression level of each gene was calculated using the $2^{-\Delta Ct}$ method [2], and presented using same type intact adipose tissue of icv saline group as 100%.

1. Cao, J.; Zhu, Q.; Liu, L.; Glazier, B. J.; Hinkel, B. C.; Liang, C.; Shi, H., Global transcriptome analysis of brown adipose tissue of diet-induced obese mice. *Int J Mol Sci* **2018,** 19, (4), 1095.
2. Livak, K. J.; Schmittgen, T. D., Analysis of relative gene expression data using real-time quantitative PCR and the 2(-Delta Delta C(T)) Method. *Methods* **2001,** 25, (4), 402-408.

1.1.1 Supplemental Material Table S1: Primers for real-time quantitative PCR.

Gene	Accession Number	Coding Protein	Primers (5′-3′)
Rpl32	P62912	ribosomal protein L32 (reference gene)	F: CATCGTAGAAAGAGCAGCAC<br>R: GCACACAAGCCATCTATTCAT
Adrb3	P26255	β3-adrenergic receptor	F: ACGCTGAGGCGCAAGAGT<br>R: GTAGGGCATATTGGAGGCAAAG
Ucp1	P04633	uncoupling protein 1	F: GGGCTGATTCCTTTTGGTCTCT<br>R: GGGTTGCACTTCGGAAGTTGT
Lipe	P15304	hormone-sensitive lipase	F: CCCCGAGATGTCACAGTCAAT<br>R: GAATTCCCGGATCGCAGAA
Pnpla2	P0C548	adipose triglyceride lipase	F: CCTGACTCGAGTTTCGGAT<br>R: CACATAGCGCACCCCTTGA
Fasn	P12785	fatty acid synthase	F: GGATGTCAACAAGCCCAAGT<br>R: CAGAGGAGAAGGCCACAAAG
Acaca	P11497	acetyl-CoA carboxylase α	F: TGAGGAGGACCGCATTTATC<br>R: GCATGGAATGGCAGTAAGGT
Gpam	P97564	glycerol-3-phosphate acyltransferase	F: GCCATCTTTGTCCACACCTT<br>R: CTCTCCGTCCTGGTGAGAAG
Dgat1	Q9ERM3	diacylglycerol acyltransferases 1	F: TGCTCTTTTTCACCCAGCTT<br>R: TTGAAGGGCTTCATGGAGTT

1.1.2. Supplemental Material Table S2: PCR products sequenced using Sanger sequencing and mapped to NCBI Blast to reveal match, gaps and mismatch.

PCR products sequenced using Sanger sequencing and mapped to NCBI Blast to reveal match, gaps and mismatch.

1. PCR product *Rpl32* (coding ribosomal protein L32): 95% match with 52/55 match, 2 gaps, and 1 mismatch.

```
Query  5    CCAATCCCCACGC-AGGCTACGCAGCGA-GAGAATGAATAGATGGCTTGTGTGCC  57
            |||||||| |||| |||||||||||||| ||||||||||||||||||||||||||
Rpl32  423  CCAATCCCAACGCCAGGCTACGCAGCGAAGAGAATGAATAGATGGCTTGTGTGCC  477
```

2. PCR product *adrb3* (coding *adrenoceptor β3*): 95% match with 39/41 match, 1 gap, and 1 mismatch.

```
Query  10   TCGCTGGTCACAGCTGACTTGGTAGTGGGACTCCTGCGTAA  50
            ||||||| ||||||||||||||||||||||||||| |||||
Adrb3  443  TCGCTGGCCACAGCTGACTTGGTAGTGGGACTCCT-CGTAA  482
```

3. PCR product *ucp1* (coding uncoupling protein 1): 89% match with 42/47 match, 1 gap, and 4 mismatches.

```
Query  15   GGTGAGTTCGACAACTTCCGAAGTGCAACCCATAAATGGGGT-AAGA  60
            ||||||||||||||||||||||||||||||||  |  ||||| ||||
Ucp1   184  GGTGAGTTCGACAACTTCCGAAGTGCAACCCACCATGGGGGTCAAGA  230
```

4. PCR product *Lipe* (coding hormone-sensitive lipase): 97% match with 35/36 match and 1 gap.

```
Query  15    CATTTTGACTCAGACCAGAAGGCATTGGGCAGTGAT  50
             |||||||||||||||||||||||||||||| |||||
Lipe   3280  CATTTTGACTCAGACCAGAAGGCATTGGGC-GTGAT  3314
```

5. PCR product *Pnpla2* (coding adipose triglyceride lipase): 98% match with 110/112 match and 2 gaps.

```
Query  7    ATATCCGCACTTTAGCT-CAAGGATGAGCTTATCCAGGCCAATGTTTGCAGCACTTTTAT  65
            |||| |||||||||||| ||||||||||||||||||||||||||||||||||||||||||
Pnpla2 504  ATAT-CGCACTTTAGCTCCAAGGATGAGCTTATCCAGGCCAATGTTTGCAGCACTTTTAT  562

Query  66   CCCTGTGTACTGTGGCCTCATTCCTCCTACCCTTCAAGGGGTGCGCTATGTG  117
            ||||||||||||||||||||||||||||||||||||||||||||||||||||
Pnpla2 563  CCCTGTGTACTGTGGCCTCATTCCTCCTACCCTTCAAGGGGTGCGCTATGTG  614
```

6. PCR product *Fasn* (coding fatty acid synthase): 91% match with 53/58 match and 5 gaps.

```
Query  16     GGGCGACCCGGGGGAAGCCTGTCCTGAGCTGGACTACTTTGCTGGCCTCTCGTCCTCT  73
              |||||||||  |||||||||||||||||||||||||||||| |||||| || ||||||
Fasn   19205  GGGCGACCC--GGGAAGCCTGTCCTGAGCTGGACTACTTTG-TGGCCT-TC-TCCTCT  19257
```

7. PCR product *Acaca* (coding acetyl-CoA carboxylase α): 90% match with 37/41 match and 4 gaps.

```
Query  13    GTTAGAGCTGA--CGGA-GAGAA-TTTTGACCTTACTGCCA  49
             |||||||||||  |||| ||||| |||||||||||||||||
Acaca  4137  GTTAGAGCTGAACCGGATGAGAAATTTTGACCTTACTGCCA  4177
```

8. PCR product *Gpam* (coding glycerol-3-phosphate acyltransferase): 95% match with 39/41 match, 1 gap, and 1 mismatch.

```
Query  14    CTGCAAAA-CTGCACAGGTACCTTCTCACCAGGACGGAGAG  53
             ||||| || ||||||||||||||||||||||||||||||||
Gapt   2186  CTGCAGAAGCTGCACAGGTACCTTCTCACCAGGACGGAGAG  2226
```

9. PCR product *Dgat1* (coding diacylglycerol acyltransferases 1): 88% match with 37/42 match, 2 gaps and 3 mismatches.

```
Query  10    GTGAAGGTTCCCTACTATCCAG-ACTCCATG-AGCCCTTCAA  49
             ||| | | |||||||||||||| |||||||| ||||||||||
Dgat1  962   GTGGATGGTCCCTACTATCCAGAACTCCATGAAGCCCTTCAA  1003
```

1.2. Results: *Adipose Tissue Gene Expression*

1.2.1 Findings

Expression of genes related to sympathetic regulation *Adrb3* and *Ucp1* in WAT and BAT was not different between saline- and BDNF-treated rats, but was significantly different between intact and denervated adipose tissues at some locations. Specifically, compared to their respective intact WAT, two-way ANOVA tests revealed that expression of *Adrb3* was significantly lower in denervated EWAT ($F = 7.26$, $p < 0.05$) and denervated RWAT ($F = 14.52$, $p < 0.001$). Multiple comparison tests indicated significant suppression of *Adrb3* expression in denervated RWAT relative to the intact RWAT of saline-treated rats ($p < 0.05$; Figure S2A). Expression of *Ucp1* in denervated BAT was significantly lower than intact BAT ($F = 14.89$, $p < 0.001$), with Tukey's *post hoc* multiple comparison test showing significant suppression of *Ucp1* expression in denervated BAT of saline-treated rats ($p < 0.01$; Figure S2B). There was no significant difference in *Ucp1* expression between intact and denervated WAT at any location (Figure S2B).

Expression of *Lipe* gene coding lipolytic enzyme HSL was significantly suppressed by denervation in BAT ($F = 24.41$, $p < 0.0001$), with Tukey's *post hoc* multiple comparison test showing significant suppression of *Lipe* expression in denervated BAT of saline-treated rats ($p < 0.01$). Expression of *Lipe* in denervated EWAT was lower than intact EWAT, but the difference did not reach statistical significance ($F = 4.09$, $p = 0.0526$; Figure S2C). Expression of *Pnpla2* gene coding lipolytic enzyme ATGL in BAT was significantly suppressed by both denervation ($F = 15.51$, $p < 0.001$) and BDNF treatment ($F = 5.52$, $p < 0.05$). Multiple comparison tests revealed that in saline-treated rats, the reduction of *Pnpla2* expression was significant in denervated BAT compared to intact BAT of saline-treated rats ($p < 0.01$; Figure S2D). Additionally, expression of *Pnpla2* gene in EWAT was also significantly lowered by denervation compared to intact EWAT, indicated by two-way ANOVA ($F = 14.39$, $p < 0.001$). Multiple comparison *post hoc* tests revealed that in saline-treated rats, the reduction of *Pnpla2* expression was significant in denervated EWAT compared to intact EWAT of saline-treated rats ($p < 0.05$; Figure 5D). Expression of neither *Lipe* nor *Pnpla2* in IWAT or RWAT was affected by denervation or BDNF treatment (Figure S2C and S2D).

Expression of lipogenic genes involved in fatty acid synthesis (*Acaca* and *Fasn*) and triglyceride synthesis (*Gpam* and *Dgat1*) in denervated BAT and EWAT was also lower compared to their respective intact adipose tissues. Specifically, expression of *Acaca* and *Fasn* related to fatty acid synthesis was significantly suppressed by denervation in BAT (*Acaca*: $F = 7.92$, $p < 0.01$; *Fasn*: $F = 12.53$, $p < 0.01$) and EWAT (*Acaca*: $F = 20.55$, $p < 0.0001$; *Fasn*: $F = 8.93$, $p < 0.01$), but not in RWAT or IWAT (Figure S2E and S2F). Expression of *Gpam* (Figure S2G) and *Dgat1* (Figure S2H) related to triglyceride synthesis was significantly suppressed by denervation in BAT (*Gpam*: $F = 5.89$, $p < 0.05$; *Dgat1*: $F = 11.45$, $p < 0.01$) and EWAT (*Gpam*: $F = 16.89$, $p < 0.001$; *Dgat1*: $F = 13.58$, $p < 0.001$). Additionally, BDNF treatment significantly reduced expression of *Gpam* in RWAT ($F = 4.23$, $p < 0.05$; Figure S2G).

1.2.2. Figure S2: Expression of genes in intact and denervated brown and white adipose tissues of intracerebroventricular (icv) saline- or BDNF-treated rats.

Expression of genes related to sympathetic regulation, including β3-adrenergic receptor (*Adrb3*; **A**) and uncoupling protein 1 (*Ucp1*; **B**); related to lipolysis, including hormone-sensitive lipase (*Lipe*; **C**) and adipose triglyceride lipase (*Pnpla2*; **D**); and related to lipogenesis, including acetyl-CoA carboxylase *α (Acaca*; **E**), fatty acid synthase *(Fasn*; **F**), glycerol-3-phosphate acyltransferase *(Gpam*; **G**), and diacylglycerol acyltransferases 1 (*Dgat1*; **H**) in intact (open bar) and denervated (filled bar) brown adipose tissues (BAT), epididymal white adipose tissue (EWAT), inguinal WAT (IWAT) and retroperitoneal WAT (RWAT) of saline- or BDNF-treated rats. Data were normalized to reference gene *Rpl32* and were presented as % of mRNA level of the same type intact adipose tissue of icv saline-treated group. Two-way ANOVA was used with the factors "innervation" (N; intact vs. denervated) and "BDNF" (B; icv saline vs. BDNF treatment) with multiple comparisons using *post hoc* Tuckey's test. * Represent significant differences relative to saline-treated rats ($p < 0.05$). † Represent significant differences relative to denervated adipose tissue ($p < 0.05$).

(A) BAT EWAT IWAT RWAT

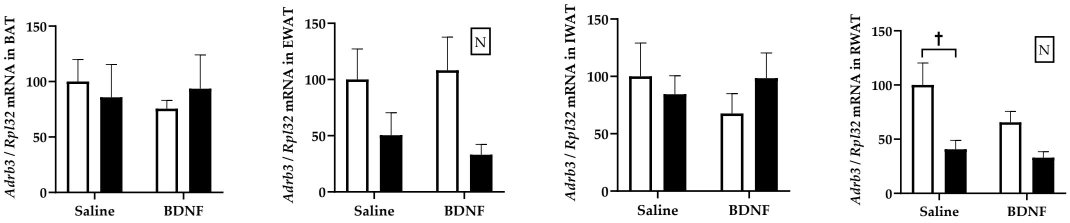

(B) BAT EWAT IWAT RWAT

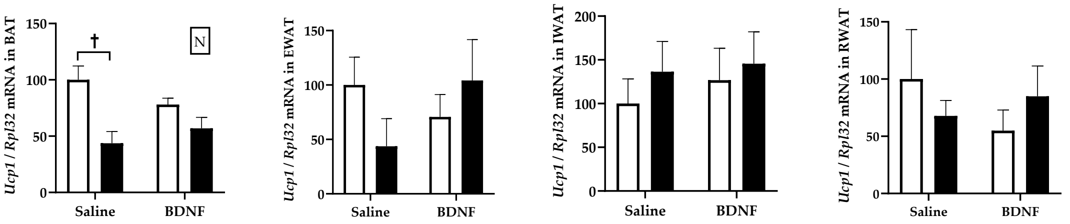

(C) BAT EWAT IWAT RWAT

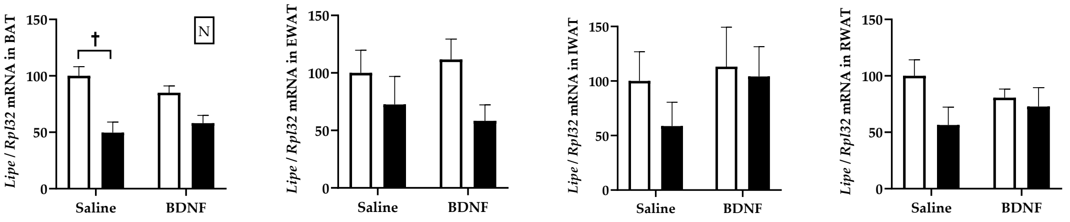

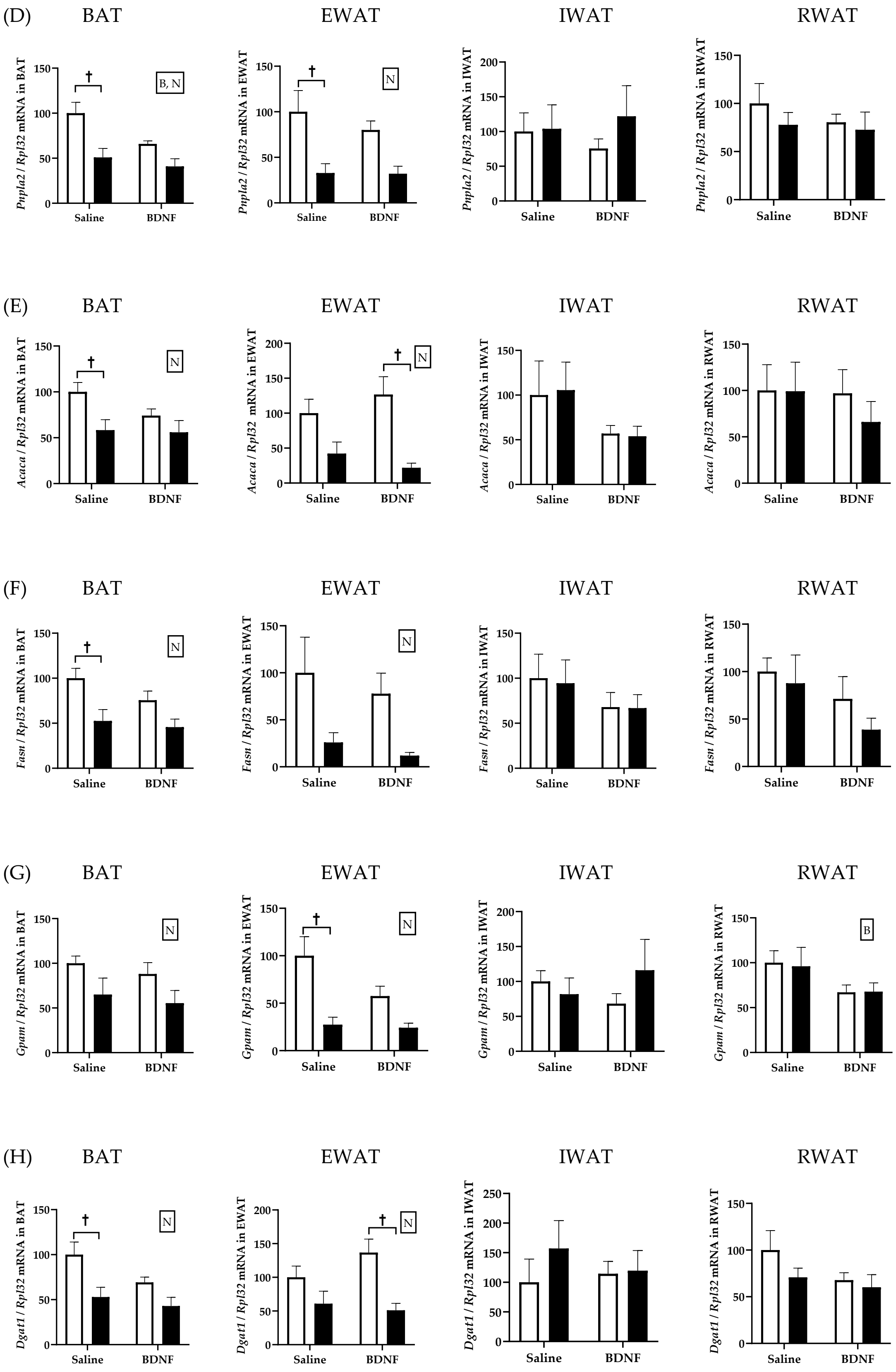

(D)
BAT
EWAT
IWAT
RWAT
Pnpla2 / Rpl32 mRNA in BAT
Pnpla2 / Rpl32 mRNA in EWAT
Pnpla2 / Rpl32 mRNA in IWAT
Pnpla2 / Rpl32 mRNA in RWAT
†
B, N
N
Saline
BDNF
(E)
BAT
EWAT
IWAT
RWAT
Acaca / Rpl32 mRNA in BAT
Acaca / Rpl32 mRNA in EWAT
Acaca / Rpl32 mRNA in IWAT
Acaca / Rpl32 mRNA in RWAT
†
N
Saline
BDNF
(F)
BAT
EWAT
IWAT
RWAT
Fasn / Rpl32 mRNA in BAT
Fasn / Rpl32 mRNA in EWAT
Fasn / Rpl32 mRNA in IWAT
Fasn / Rpl32 mRNA in RWAT
†
N
Saline
BDNF
(G)
BAT
EWAT
IWAT
RWAT
Gpam / Rpl32 mRNA in BAT
Gpam / Rpl32 mRNA in EWAT
Gpam / Rpl32 mRNA in IWAT
Gpam / Rpl32 mRNA in RWAT
†
N
B
Saline
BDNF
(H)
BAT
EWAT
IWAT
RWAT
Dgat1 / Rpl32 mRNA in BAT
Dgat1 / Rpl32 mRNA in EWAT
Dgat1 / Rpl32 mRNA in IWAT
Dgat1 / Rpl32 mRNA in RWAT
†
N
Saline
BDNF

Supplementary Materials

2. Supplemental Material Adipose Tissue Norepinephrine (NE) Concentration

2.1. Results: Adipose Tissue NE Concentration

Concentration of sympathetic neurotransmitter NE from ip vehicle (no αMPT) groups (*i.e.* $[NE]_0$) was very different between various WAT and BAT of saline-treated rats (F = 8.518, $P < 0.001$; Figure S1A). Multiple comparison tests revealed that NE concentration was significantly higher in intact BAT (1.1362 ± 0.3600 ng/mg) than intact EWAT (0.0336 ± 0.0075 ng/mg), IWAT (0.1338 ± 0.0488 ng/mg), and RWAT (0.0651 ± 0.0191 ng/mg) ($p < 0.01$). In contrast, NE concentration was not significantly different among different types of WAT (Figure S1A). When NE concentration was compared between intact and denervated same type adipose tissues, except for denervated BAT (F = 2.60, $p > 0.05$; Figure S1B), NE concentration was significantly affected by innervation, with denervated EWAT (F = 8.24, $p < 0.01$; Figure S1C), IWAT (F = 4.25, $p < 0.05$; Figure S1D), and RWAT (F = 11.16, $p < 0.01$; Figure S1E) having significantly lower NE concentration than their respective intact adipose tissues. Central injection of BDNF, however, did not affect NE concentration of any intact or denervated adipose tissues ($p > 0.05$; Figure S1).

2.2. Supplemental Material Figure S1: Norepinephrine content in intact and denervated brown and white adipose tissues of intracerebroventricular (icv) saline- or BDNF-treated rats.

Baseline norepinephrine concentration $[NE]_0$ in intact brown adipose tissues (BAT), epididymal white adipose tissue (EWAT), inguinal WAT (IWAT), and retroperitoneal WAT (RWAT) of icv saline-treated rats that received intraperitoneal injection of vehicle (**A**). One-way analysis of variance (ANOVA) was used to compare $[NE]_0$ of intact adipose tissues in saline-treated rats (**A**). $[NE]_0$ in intact (open bar) and denervated (filled bar) BAT (**B**), EWAT (**C**), IWAT (**D**), and RWAT (**E**) of icv saline- or BDNF-treated rats that received intraperitoneal injection of vehicle. Two-way ANOVA was used with the factors "innervation" (N; intact vs. denervated) and "BDNF" (B; icv saline vs. BDNF) and multiple comparisons *post hoc* Tuckey's test (**B-E**). # Represent significant differences relative to BAT ($p < 0.05$). † Represent significant differences relative to denervated adipose tissue ($p < 0.05$).

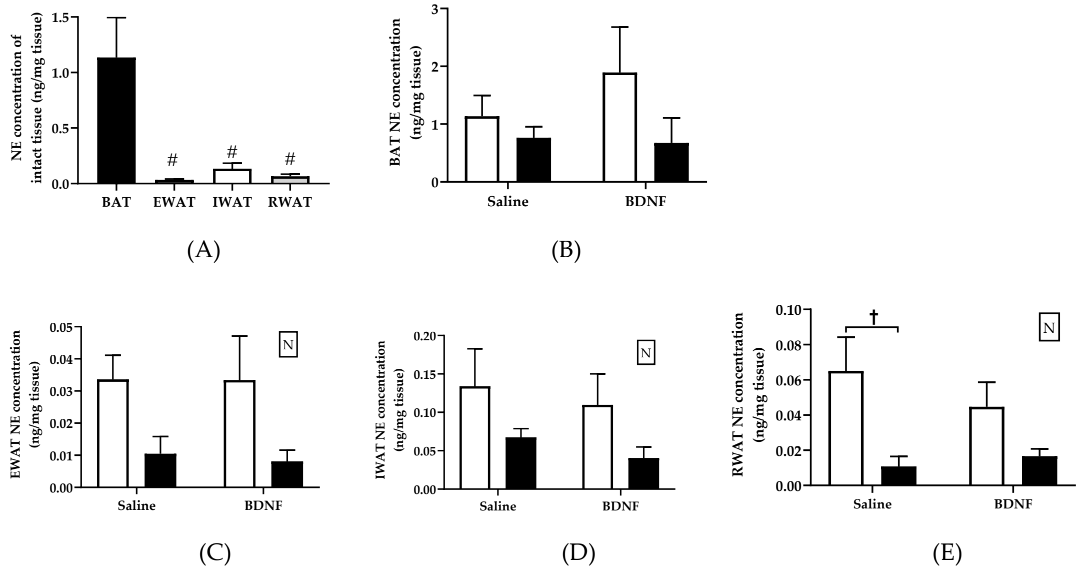

Supplement: Supplementary file 1 [file biomolecules-09-00452-s001.pdf]
